# Weak Ultrasound Contributes to Neuromodulatory Effects in the Rat Motor Cortex

**DOI:** 10.3390/ijms24032578

**Published:** 2023-01-30

**Authors:** Po-Chun Chu, Chen-Syuan Huang, Pi-Kai Chang, Rou-Shayn Chen, Ko-Ting Chen, Tsung-Hsun Hsieh, Hao-Li Liu

**Affiliations:** 1 Department of Electrical Engineering, National Taiwan University, Taipei 10617, Taiwan; 2 Department of Pediatrics, School of Medicine, University of Utah, Salt Lake City, UT 84112, USA; 3 Division of Movement Disorder, Department of Neurology, Chang Gung Memorial Hospital and Chang Gung University College of Medicine, Taipei 10507, Taiwan; 4 Department of Neurosurgery, Chang Gung Memorial Hospital at Linkou, Taoyuan 33305, Taiwan; 5 Neuroscience Research Center, Chang Gung Memorial Hospital, Linkou, Taoyuan 33305, Taiwan; 6 School of Physical Therapy, Graduate Institute of Rehabilitation Science, Chang Gung University, Taoyuan 33302, Taiwan; 7 Healthy Aging Research Center, Chang Gung University, Taoyuan 33302, Taiwan

**Keywords:** transcranial focused ultrasound, neuromodulation, weak ultrasound, rats, motor-evoked potentials, transcranial magnetic stimulation, in vivo, c-fos, GABAergic neurons, PIEZO-1 protein

## Abstract

Transcranial focused ultrasound (tFUS) is a novel neuromodulating technique. It has been demonstrated that the neuromodulatory effects can be induced by weak ultrasound exposure levels (spatial-peak temporal average intensity, I_SPTA_ < 10 mW/cm^2^) in vitro. However, fewer studies have examined the use of weak tFUS to potentially induce long-lasting neuromodulatory responses in vivo. The purpose of this study was to determine the lower-bound threshold of tFUS stimulation for inducing neuromodulation in the motor cortex of rats. A total of 94 Sprague–Dawley rats were used. The sonication region aimed at the motor cortex under weak tFUS exposure (I_SPTA_ of 0.338–12.15 mW/cm^2^). The neuromodulatory effects of tFUS on the motor cortex were evaluated by the changes in motor-evoked potentials (MEPs) elicited by transcranial magnetic stimulation (TMS). In addition to histology analysis, the in vitro cell culture was used to confirm the neuromodulatory mechanisms following tFUS stimulation. In the results, the dose-dependent inhibitory effects of tFUS were found, showing increased intensities of tFUS suppressed MEPs and lasted for 30 min. Weak tFUS significantly decreased the expression of excitatory neurons and increased the expression of inhibitory GABAergic neurons. The PIEZO-1 proteins of GABAergic neurons were found to involve in the inhibitory neuromodulation. In conclusion, we show the use of weak ultrasound to induce long-lasting neuromodulatory effects and explore the potential use of weak ultrasound for future clinical neuromodulatory applications.

## 1. Introduction

Neuromodulation is currently a rapidly growing field where technology refers to interacting and interfering with the nervous system through chemical, mechanical, electrical, or electromagnetic methods to activate, inhibit, regulate or modify the neural activity for the treatment of neurological and neuropsychiatric disorders [1,2,3]. Neuromodulatory techniques have been developed through invasively or non-invasively stimulating cortical or subcortical regions for modulating brain activity [4,5,6]. For example, as a clinical tool, the deep brain stimulation technique (DBS) is a typical invasively intracranial neuromodulation approach that requires the surgical implantation of stimulating electrodes to deep brain area and has been proven to be effective for Parkinson’s disease, essential tremor, dystonia, and epilepsy [7,8,9,10,11]. Non-invasive neuromodulation approaches such as repetitive transcranial magnetic stimulation (rTMS) or transcranial direct current stimulation (tDCS), are capable of inducing after-effects outlasting the stimulation period through plasticity-like mechanisms and have been approved for therapeutic purposes in neurological and psychiatric disorders [1,12,13]. However, the rTMS or tDCS have major limits. For example, the stimulation areas of rTMS or tDCS are relatively superficial and cannot penetrate deeper than the cortical layer of the brain, and their effects are variable both within and between individuals [13,14,15]. Hence, there is an unmet need for a novel transcranial brain stimulation technique for modulating a deeper area and for more consistent effects.

Transcranial focused ultrasound (tFUS), which converges the acoustic wave of ultrasound to a target point distant from the transducer, is a novel neuromodulating technology for noninvasively targeting deep brain tissue [4,16,17]. In particular, the main advantage is that the tFUS can stimulate much deeper brain structures and has a higher spatial resolution than rTMS or tDCS [18]. In contrast to high-intensity focused ultrasound which has shown its success in tissue ablation through a thermal effect, tFUS at a much lower intensity has shown the ability to modulate neuronal activity without causing tissue damage [19,20,21,22,23]. For instance, following ultrasound stimulation, suppressive neuromodulatory effects on human motor cortical excitability as measured by motor-evoked potentials (MEPs) elicited by transcranial magnetic stimulation (TMS) were found [24]. Another study demonstrated that tFUS delivered transiently increased excitability in the motor cortex [25]. The alternation of main effects from the facilitation or inhibition induced by tFUS could result from the application of differing tFUS parameters, such as duty cycle, amplitude, or duration of stimulation [26,27].

Concerning stimulation intensity of tFUS for neuromodulation, several in vivo and human studies utilized different exposure levels of tFUS from tens to thousands of milliwatts per square centimeter for identifying the neuromodulatory effects. Wang et al. [28] and Yuan et al. [29] found an increase in neural oscillations and cortical hemodynamic responses in the motor cortex by using tFUS with 80 to 400 mW/cm^2^ spatial-peak temporal-average intensity (I_SPTA_). In the animal study, Kim and associates demonstrated the suppressive responses to the sonication of either the primary motor cortex or the thalamus in conscious sheep at 3600 mW/cm^2^ I_SPTA_ tFUS [30]. Dallapiazza et al. showed that 25,000 mW/cm^2^ I_SPTA_ tFUS could functionally modulate to somatosensory evoke potential while delivering energy to the swine thalamus [21]. The findings of animal and human experiments have promoted the understanding of different biological effects based on different exposure levels of tFUS and show that the intensity of tFUS could be a key parameter for the induction of neuromodulation.

Recently, in contrast to the high intensity of tFUS for the neuromodulation, the application of weak-intensity (I_SPTA_ < 10 mW/cm^2^) ultrasound to induce a neural modulatory effect has been reported in vitro and brain-slice experiments [31,32]. However, a physiological response from neuromodulation after weak-intensity sonication has not been demonstrated. To our knowledge, no study has directly investigated the cortex of the in vivo brain. We hypothesize that an in vivo and long-lasting neuromodulating effect can be generated by using weak ultrasound in a transcranial noninvasive manner. For this reason, a feasibility study of using weak ultrasound (I_SPTA_ < 10 mW/cm^2^) to explore neuromodulatory effects is needed. The purpose of this study is trying to explore the feasibility to evoke the neuromodulation effects via designed increased intensity level of weak ultrasound (I_SPTA_ < 10 mW/cm^2^) and with the assessment of motor-evoked potentials (MEPs) in the primary motor cortex (M1) of rat brains [24]. We also additionally assessed the selected ultrasound parameters with real-time calcium signals from primary neurons and histologic examinations to identify potential physiological mechanisms.

## 2. Results

The neuromodulatory effects of tFUS were evaluated by the changes of MEPs elicited by TMS on the motor cortex (M1) of rat brains [24,33,34]. Furthermore, to evaluate the safety of tFUS and verify the neuronal activity changes following weak tFUS, the glial fibrillary acidic protein (GFAP) staining and expression levels of c-Fos and GAD-65 were conducted. Furthermore, to identify potential physiological mechanisms, we additionally assessed the selected ultrasound parameters with real-time calcium signals in vitro from primary neurons through real-time calcium signal and immunostaining with the piezo inhibitor to confirm the activities of modulated GABAergic neurons.

### 2.1. The Changes in Motor-Evoked Potentials (MEPs) before and after Weak Sonication

**Figure 1:**
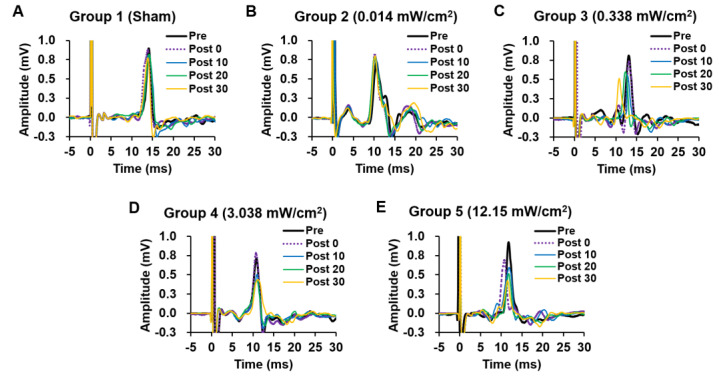
Representative MEPs of a rat before and after sham stimulation with various intensities of tFUS are presented at each measured period (preintervention and 0, 10, 20, 30 min after tFUS) with a colored line. (**A**) The MEPs of the right limb for each time point after sham treatment (**B**) The MEPs of the right limb for each time point after I_SPTA_ of 0.014 mW/cm^2^ (**C**) The MEPs of the right limb for each time point after I_SPTA_ of 0.338 mW/cm^2^ (**D**) The MEPs of the right limb for each time point after I_SPTA_ of 3.038 mW/cm^2^ (**E**) The MEPs of the right limb for each time point after I_SPTA_ of 12.15 mW/cm^2^.

To evaluate the neuromodulatory effects after ultrasound stimulation, animals were anesthetized and then mounted in a prone position in a stereotaxic apparatus. A homemade 0.7-MHz focused ultrasound transducer was used to deliver spatial-peak temporal average intensity (I_SPTA_) from 0.014 mW/cm^2^ to 50 mW/cm^2^ for 5 min. MEPs were recorded for 10 min before tFUS to serve as the baseline, then MEP recording continued for 30 min to follow the post-tFUS effect. All animals exhibited 70.25 ± 1.16 (mean ± SEM) maximum machine output in resting motor threshold (RMT), and no significant differences between groups were observed (Supplementary Figure S1). Figure 1 illustrates a typical case of MEP change after various intensities of weak tFUS (from I_SPTA_ 0 mW/cm^2^ (sham) to I_SPTA_ 12.15 mW/cm^2^). The MEPs of the right limb for each time point after sham treatment (group 1) and I_SPTA_ of 0.014 mW/cm^2^ exhibited no significant changes (Figure 1A,B). The MEPs decreased 10 min after I_SPTA_ of 0.338 mW/cm^2^ and I_SPTA_ of 3.038 mW/cm^2^ were applied (Figure 1C,D). Notably, following ultrasound exposure at I_SPTA_ of 12.15 mW/cm^2^, the amplitude of MEPs decreased to 0.5 mV and persisted under that low level for 30 min (Figure 1E).

**Figure 2:**
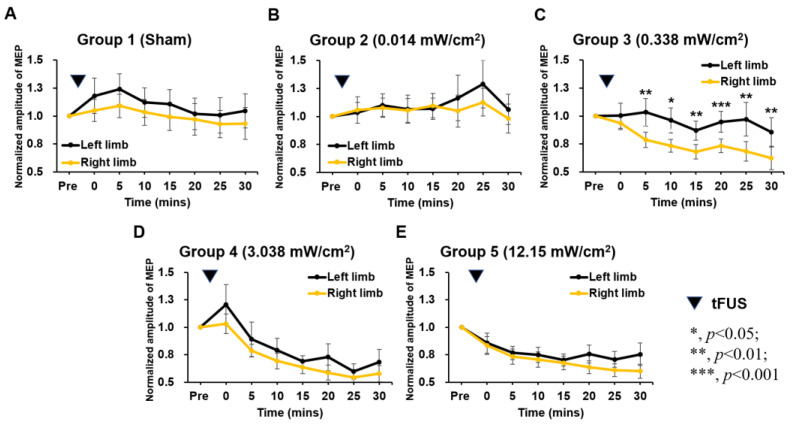
Comparison of the averaged MEP from both limbs in various groups 30 min after tFUS (*, **, and *** represent *p* values of <0.05, <0.01, and <0.001, respectively).

We quantitatively evaluated the neuromodulation effect in each group by observing MEP responses in both limbs. Increased exposure levels monotonically decreased MEP responses (Figure 2). The normalized averaged amplitude of MEP data for left/right limbs from group 1 (sham) and group 2 (I_SPTA_ 0.014 mW/cm^2^) exhibited no significant difference (Figure 2A,B). Following exposure to an I_SPTA_ of 0.338 mW/cm^2^ (group 3, Figure 2C), the amplitude of MEPs for the right limb decreased from 0.94 ± 0.22 to 0.79 ± 0.28, which was significantly lower than the MEP for the left limb (from 1.00 ± 0.44 to 0.83 ± 0.49, *p* < 0.05 at all time points). Over the post-tFUS period (0–30 min), group 4 (I_SPTA_ 3.038 mW/cm^2^) and group 5 (I_SPTA_ 12.15 mW/cm^2^) exhibited declined MEPs of the right limb (30.5% and 31.4% decreases compared with pre-FUS values, respectively) (Figure 2D,E). In group 4 and group 5, the MEPs of the left limb also decreased by 20% and 24.3%, respectively, but no significant differences were observed between limbs (*p* = 0.353 and 0.356, respectively). This indicates that the higher intensity of tFUS (I_SPTA_ > 3.038 mW/cm^2^) can lead to a suppression effect in not only the ipsilateral sonicated motor cortex but also in the contralateral region.

**Figure 3:**
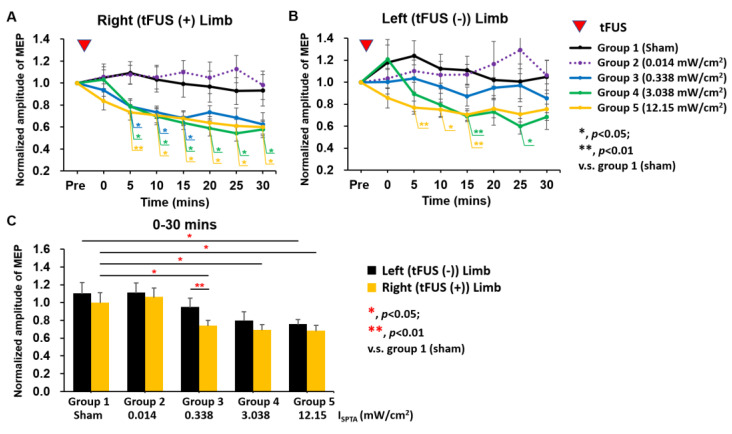
Overall changes in MEP in all groups. (**A**) Changes in right limbs (tFUS targeted) within 30 min after tFUS (**B**) Change in left limbs (tFUS untargeted) within 30 min after tFUS. (**C**) Change in both limbs at overall follow-up. (* and ** represent *p* values of <0.05 and <0.01, respectively).

To compare the neuromodulatory effect among various sonication groups, 30-min longitudinal MEP responses were rearranged and colocalized (Figure 3). For the MEPs in the targeted limb, repeated-measures ANOVA indicated the significant main of time (F_7,525_ = 14.104, *p* < 0.001), ultrasound exposure dose (F_4,75_ = 5.129, *p* = 0.001), and time × dose interaction (F_28,525_ = 2.609, *p* < 0.001). The MEPs of both limb at each time point after sham tFUS (group 1) and tFUS I_SPTA_ 0.014 mW/cm^2^ exhibited no significant changes (Figure 3A,B). At 30 min post-tFUS, tFUS reduced the MEP amplitude of the targeted limb as the intensity exceeded an I_SPTA_ of 0.338 mW/cm^2^ (Figure 3A), but intensities of only 3.038 and 12.15 mW/cm^2^ could be observed on the left side, suggesting that the ultrasound-exposure-induced neural suppression effect is target-side-dependent (Figure 3B). Conversely, differences in MEP amplitude changes in the untargeted limb were noticeable only in groups 4 (I_SPTA_ 3.038 mW/cm^2^) and 5 (I_SPTA_ 12.15 mW/cm^2^), which were observed 5–15 min after tFUS (*p* < 0.05, compared to group 1 (sham)). Similarly, changes in MEP amplitude did not recover to baseline levels in these two groups, indicating that the duration of neuromodulation depended on tFUS intensity. In terms of overall time (0–30 min), MEP amplitudes of 0.338 W/cm^2^ (group 3) began to induce an MEP-suppressive effect in the targeted motor cortex (no effect was observed on the untargeted contralateral region), which appears to be the minimal exposure level required to induce a neuromodulatory suppressive effect (Figure 3C).

### 2.2. Histological Examinations

**Figure 4:**
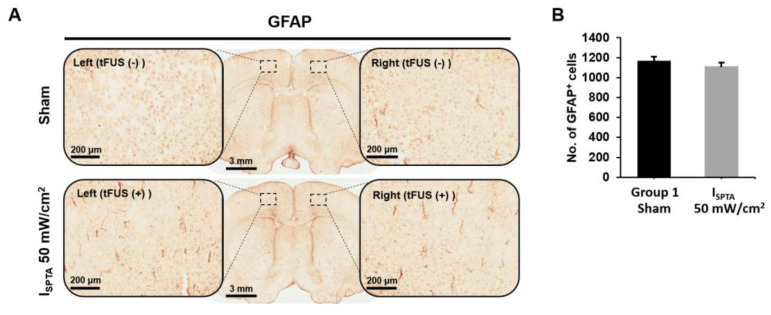
(**A**) Representative images of glial fibrillary acidic protein (GFAP) immunostaining and examples of GFAP images after sham tFUS and tFUS at an I_SPTA_ of 50 mW/cm^2^. (**B**) No significant difference was observed between the two groups in the GFAP of the right hemisphere.

To evaluate whether tFUS induces neuron damage, rats were sacrificed 90 min after tFUS at an I_SPTA_ of 50 mW/cm^2^. The GFAP staining was conducted, and group 1 (sham) and animals that received tFUS at an I_SPTA_ of 50 mW/cm^2^ were used as a comparison group. Upon GFAP immunohistochemical staining, no obvious astrogliosis at or near the sonicated sites in brains treated with tFUS was observed (Figure 4A). Quantification analysis demonstrated no significant change in GFAP immunoreactivity after I_SPTA_ = 50 mW/cm^2^ sonication compared with group 1 treatment (sham; t = 1.066, *p* = 0.328) (Figure 4B). This suggests that the tFUS intensities employed in this study do not affect the normal phenotype of astrocytes in rat brains.

**Figure 5:**
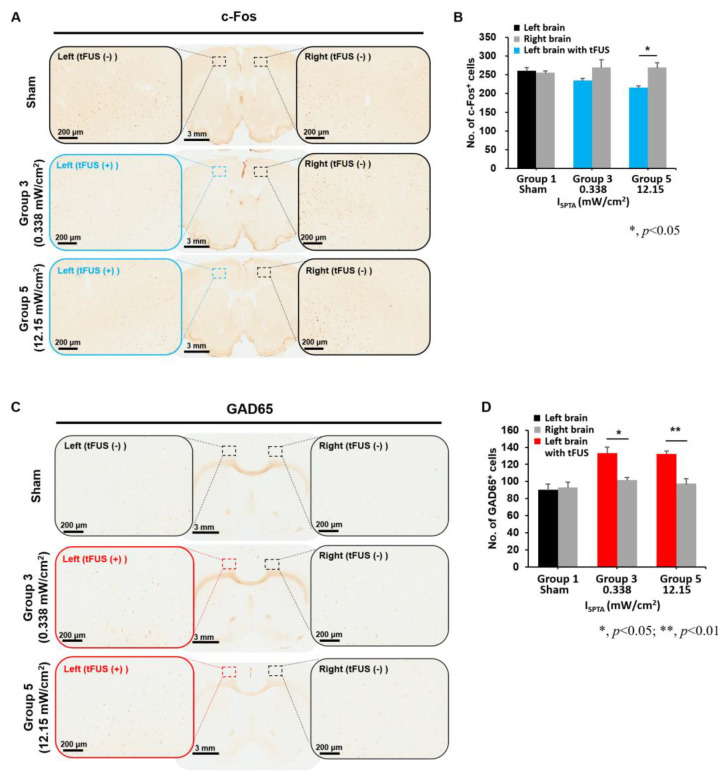
Comparison of c-Fos and GAD-65 signals in motor cortexes among the tested groups, including groups 1 (sham), 3, and 5. (**A**) A typical example of c-Fos staining (**B**) c-Fos-positive cell change in group 1 (sham), group 3 (I_SPTA_ = 0.338 mW/cm^2^), and group 5 (I_SPTA_ = 12.15 mW/cm^2^). Blue windows and bars represent the effects of tFUS. (**C**) A typical example of GAD-65 staining (**D**) GAD-65-positive cell changes in group 1 (sham), group 3 (I_SPTA_ = 0.338 mW/cm^2^), and group 5 (I_SPTA_ = 12.15 mW/cm^2^). Red windows and blue bars represent the effects of tFUS. (* and ** represent *p* values of <0.05 and <0.01, respectively).

We further verified the expression levels of c-Fos and GAD-65 as markers of neuronal and synaptic activity to verify neuronal activity changes induced using weak ultrasound in two representative groups (group 3 [I_SPTA_ = 0.338 mW/cm^2^] and group 5 [I_SPTA_ = 12.15 mW/cm^2^]) (Figure 5). Compared with c-Fos markers of the contralateral brain in group 3 and group5, animals receiving tFUS at an I_SPTA_ of 0.338 mW/cm^2^, exhibited a greater reduction in c-Fos-positive neurons (261 ± 13.7 to 235 ± 9.4 cell counts in the ipsilateral motor cortex versus sham FUS. tFUS at an I_SPTA_ of 12.15 mW/cm^2^ further decreased c-Fos-positive neurons (215 ± 8.8 cell counts in the ipsilateral motor cortex versus sham FUS t = 4.87, *p* = 0.008). No reduction in cell counts in the contralateral cortex was observed). The animals with sham tFUS stimulation had a symmetric c-Fos-positive signal between hemispheres, indicating that weak tFUS reduced neuronal activity in the targeted motor cortex.

According to a GAD-65 analysis, animals that received sham tFUS had symmetric positive cells in the ipsilateral and contralateral motor cortexes (93 ± 11.3 versus 92 ± 7.1, t = 0.4, *p* = 0.71). Exposure to tFUS of 0.338 mW/cm^2^ strongly increased GAD-65-positive cell counts compared with those in the contralateral motor cortex (133 ± 11.85 versus 102 ± 5.4, t = 4.2, *p* = 0.014). Similarly, further increased GAD-65 cell counts (t = 5.9, *p* = 0.004) were observed in the ipsilateral motor cortex after tFUS at 12.15 mW/cm^2^ (131 ± 3.5 versus 95 ± 9.5 cell counts, respectively), indicating weak tFUS-induced GABA inhibitor generation in the sonicated area, and an ultrasound dose-dependent pattern was observed.

### 2.3. Primary Neuron Cells with Weak Sonication

**Figure 6:**
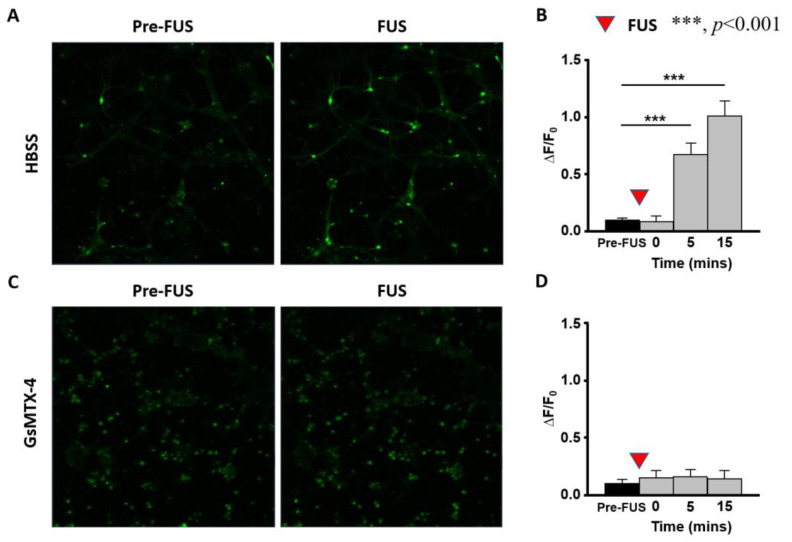
Calcium images of primary neurons following weak ultrasound sonication (I_SPTA_ = 12.15 mW/cm^2^). (**A**) Representative images of Fluo-4-AM fluorescence presenting calcium signals in primary neurons before and after weak sonication. (**B**) Quantitative analysis indicates that weak sonication increases the levels of calcium signals in primary neurons within 20 min. Bars represent mean ± SD from two experiments. (n = 66, *** represents *p* < 0.001, Friedman test post hoc Tukey test). (**C**) Representative calcium images of primary neurons pretreated with 5 μM GsMTx-4 before and after weak sonication. (**D**) Quantitative analysis indicated no increased calcium signals in primary neurons pretreated with GsMTx-4 after weak sonication within 20 min. Bars represent the mean ± SD from three experiments. (n = 219. *** represents *p* < 0.001, one-way repeated-measures analysis post hoc Tukey test). ΔF/F0: fluorescence changes/before weak sonication.

To confirm the ultrasound-induced neuromodulatory pathway, calcium exchange caused by neural activity change, interfered with by ultrasound, was then observed in vitro from the primary neurons obtained from embryonic rodent cerebral cortices. The signal of calcium influx induced by weak ultrasound exposure (I_SPTA_ = 12.15 mW/cm^2^) was recorded (Figure 6A), which was observed using the detected green fluorescence with the Fluo-4 calcium indicator. An increasing amplitude of calcium influx was detected from 0.09 ± 0.02 to 1 ± 0.13 15 min after 5-min sonication, suggesting weak-intensity ultrasound activated the calcium channels of primary neurons (Figure 6B). Images from Fluo-4 staining and anti-GABA staining were used to determine the subtype of ultrasound-activated neurons. We observed that GABAergic neurons had anti-GABA positive signals and also expressed Fluo-4-positive signals, suggesting that inhibitory type neurons were stimulated by weak sonication (Supplementary Figure S2), providing evidence to confirm the in vivo observation that weak-intensity ultrasound induced inhibitory neuromodulatory effect.

Figure 6C,D demonstrates that the inhibitor GsMTX-4 of piezo channels blocked calcium signal activation from ultrasound weak exposure caused by the addition of HBSS-medium. This suggests that the PIEZO-1 proteins of GABAergic neurons are highly involved in the cortical inhibitory neuromodulation process evoked by weak-intensity ultrasound.

## 3. Discussion

In the current study, we confirmed that weak ultrasound can induce neuromodulation effects in rat motor cortical regions and identified its major modulation types. In addition to the electrophysiological examination, the immunohistochemistry examinations in c-Fos, GAD-65, and primary neuron calcium quantification were conducted to confirm the ultrasound-induced neuromodulatory effects under weak exposure.

We have established a tFUS-TMS rat model to identify the neuromodulatory effects induced by weak tFUS exposure by measuring TMS-evoked MEPs in rats. We found that dose-dependent neuromodulation effects on the motor cortex were induced by different exposure levels of tFUS. We found that 5 min tFUS (I_SPTA_ of 0.338–12.15 mW/cm^2^) decreased the amplitude of MEPs for 30 min, whereas the low intensity of tFUS (I_SPTA_ of 0.014 mW/cm^2^) did not inhibit MEPs. Based on the results, we determined that ultrasound exposure levels as low as 0.338 mW/cm2 can induce neuromodulatory effects and may be the lower-bound exposure threshold. Although earlier studies have reported similar ideas in vitro [31,35], to our best knowledge, this work is the first to test the feasibility of using weak ultrasound exposure to induce the neuromodulatory effects in vivo. The findings of this study may also provide useful information for exploring the potential of using weak ultrasound stimulation for future clinical applications such as neuromodulation therapy for epilepsy, Parkinson’s disease, essential tremors, depression, or neuropathic pain [18,36,37,38,39].

The current results confirm that tFUS pulsations induce neural excitatory (c-Fos, activation of cells) and inhibitory (GAD-65) synaptic transmission, indicating that the neuromodulatory effect induced by weak ultrasound stimulation originated in the inhibitory-type neurons. On the basis of increased GAD-65 expression and reduced c-Fos expression after weak ultrasound stimulation, the results reveal that weak tFUS exposure can generally suppress the neuron activity in the motor cortex. The decrease in c-Fos-positive cells and a strong increase in the GAD-65 signal indicated the deactivation of cortical activity and an increase in the activity of inhibitory interneurons. Similar results were also reported in the previous tFUS-epilepsy study, showing the decreased expression of c-Fos and increased GAD-65 levels in the cortex and hippocampus [37]. Furthermore, the finding of a strengthening of the GABAergic synapses by tFUS is supported by another noninvasive repetitive TMS (rTMS) study, showing that GAD-65 was increased following rTMS and demonstrated that rTMS affected the expression of activity-dependent proteins in cortical inhibitory interneurons [40]. Although the precise mechanism underlying the the tFUS induced neuromodulation effects remains unclear, our current results suggest that the activation in inhibitory neurons could play a critical role in the tFUS induced neuromodulation.

We further observed that weak (I_SPTA_ < 10 mW/cm^2^) ultrasound significantly increased the calcium influxes in primary neurons, which responded to the increased Fluo-4 staining on GABAergic neurons. Although the detailed mechanisms of weak ultrasound–induced neuromodulatory effects have not yet been fully explored, earlier in vitro studies utilized low-intensity (I_SPTA_ < 100 mW/cm^2^) showed that the calcium influx in primary cortical neurons is mediated from the specific mechanosensitive ion channels after ultrasound stimulation [41,42]. The mechanosensitive channels such as transient receptor potential channels, acid-sensing ion channels (ASICs), and Piezo channels were reported to play a role in mechanosensitive interaction with the aforementioned acoustic regimes [43,44]. A previous cell culture study reported that ASICs and cytoskeletal proteins induce a rapid calcium flow change (a few seconds after sonication) under weak ultrasound exposure (I_SPTA_ < 10 mW/cm^2^) [31]. Unlike the rapid response of ASIC proteins, we found that the piezo-type mechanosensitive ion channels (e.g., PIEZO-1) exhibited relatively slow dynamics (with minutes after sonication). Because the observed neural modulatory dynamics in this study did not exhibit a rapid response pattern, it is suggested that the triggered proteins in this study were mechanosensitive ion channel proteins. Further investigations are required to provide definitive evidence supporting this hypothesis.

Considerable attention has been paid to the duration of neuromodulatory effects induced by tFUS. In the current study, the results show that 5 min tFUS can inhibit motor cortical excitability for 30 min. When compared with other studies, Kim et al. reported a reversely suppressive effect for 2 min in the motor cortex with 1-min tFUS [30], and another study reported that 40-s tFUS had a lasting effect on the supplementary motor area or frontal polar cortex in primates, producing even longer-lasting modulation of brain cortical activation and connectivity (>1 h) [45]. Yoon et al. reported a 5-min neural suppression effect in the somatosensory cortex after a 2-min sonication [46]. Dallapiazza and colleagues used 40-s tFUS to suppress somatosensory evoked potential in a swine brain for 5 min [21]. In a rabbit model, Yoo et al. reported that 18-s tFUS to the visual cortex suppressed visual evoked potential for 15 min [47]. tFUS suppressed pharmacal-induced epileptic signals for 30 min more than 1 week after 10-min tFUS [37] and 30-min FUS [48]. These results suggest that the suppression effect of tFUS may be longer than the exposure duration, and this long-term effect may depend on the exposure site and total exposure duration. Based on our results, we observed that 5-min weak tFUS reduced cortical excitability for up to 30 min. A procedure involving repeated 5-min sonication or prolonged sonication may help treat chronic conditions such as Parkinson’s disease or epilepsy [49,50]

Regarding the interhemispheric neuromodulatory inhibition between ipsilateral and contralateral M1, the suppressive neuromodulation effects on the ipsilateral and contralateral M1 were found while increased tFUS intensity over 3 mW/cm^2^ (group 4, 5). tFUS-altered functional connectivities have been reported through functional magnetic resonance imaging in a primate study [45]. The possible explanation for this bilateral effect is that ultrasound mechanical whole-brain vibration may have confounding auditory effects [51]. However, the weak tFUS protocols utilized in the current study were using pulsed mode and low pulse repetition frequency [52]. The confounding auditory effect can be eliminated in the current study. Further study could be needed to find the possible mechanisms in the interhemispheric neuromodulatory inhibition induced by tFUS.

Compared with other non-invasive brain neuromodulatory tools such as rTMS and tDCS, the weak-intensity tFUS (I_SPTA_ < 10 mW/cm^2^) applied in the current study may have more clinical opportunities. We demonstrate the promising neuromodulatory effects by using a very low dose of tFUS in the motor cortex. For current FDA-approved guidelines, the clinical diagnosis ultrasound such as Transcranial Doppler (TCD) (I_SPTA_ ≤ 720 mW/cm^2^) may play a role in the application of neuromodulation. A review article shows that TCD presents several advantages including its high spatial resolution, easy administration and better clinical accessibility [53]. With delivering weak-intensity ultrasound from a diagnosis transducer, the tFUS can apply not only as diagnostic imaging but also can be a therapeutic tool for neurological disorders.

For future clinical applications of tFUS for neuromodulation, it could have a few limitations. For example, we did not characterize the long-term effects of tFUS in motor cortical excitability and neuroactivity over days. Another limitation is that the protocols of weak ultrasound delivered by the current animal setting would be needed to be modified for the future use of tFUS in humans. The adjustments of dose and related instruments of tFUS would be necessary. Despite these limitations, our findings indicate that the neuromodulatory effects induced by weak-intensity tFUS may serve as a reference for future clinical neuromodulatory studies.

In conclusion, we confirmed that weak ultrasound had long-lasting neuromodulatory effects in the motor cortical region of rats. A lower-bound threshold (I_SPTA_ 0.338 mW/cm^2^) of tFUS to induce the suppressive neuromodulatory effect, which lasted for 30 min, was identified. The findings from histology and primary neuron cells confirmed that GABA synthesis in inhibitory neurons was stimulated artificially. This study provides the safety and feasibility of the use of weak ultrasound exposure to induce neuromodulatory effects, which can explore the potential use of weak ultrasound for future clinical neuromodulatory applications in neurological and neuropsychiatric disorders.

## 4. Materials and Methods

### 4.1. Animals and Preparations

All animal experiments were approved by the Institutional Animal Care and Use Committee of Chang Gung University (IACUC No. CGU107-231). Ninety-four male Sprague-Dawley rats (350–400 g; BioLASCO, Taipei, Taiwan) were used in the experiments. The rats were housed with a 12-h light/dark cycle at a temperature of 25 ± 1 °C and had ad libitum access to food and water. All animals were initially anesthetized with Zoletil (65 mg/kg; Vibac, Carros, France) and Rompun (10 mg/kg; Bayer, Leverkusen, Germany) and then mounted on a stereotaxic apparatus (Stoelting, Wood Dale, IL, USA). The body temperature of the experimental rats was kept constant using a circulating water heater. All animals were randomly assigned into groups for testing the effects of tFUS.

### 4.2. tFUS Setup and Study Design

**Figure 7:**
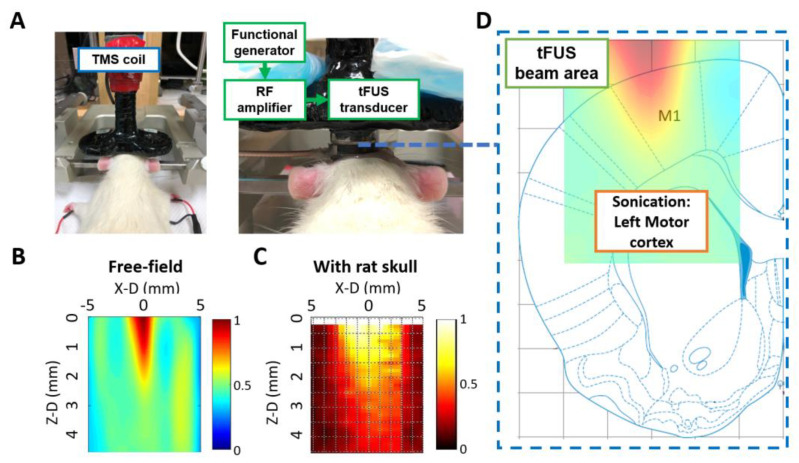
(**A**) The Center of the transcranial magnetic stimulation (TMS) coil and transcranial focused ultrasound (tFUS) transducer are positioned and focused on the motor cortex of the left hemisphere. (**B**) Characterization of the FUS pressure fields in sagittal (Y–Z plane) scans of ultrasound pressure distribution using a hydrophone-based ultrasound field mapping system. (**C**) Characterization of the tFUS pressure fields in sagittal (Y–Z plane) scans. (**D**) Conceptual schematic of the topographical sonication depth targeting the motor cortex area [56].

The anesthetized test rats were mounted on a stereotaxic apparatus 60 min before focused ultrasound (FUS) to avoid the dampening of neuronal activity from anesthesia (Figure 7A). Anesthesia depth was adjusted for the absence of abdominal contractions to the tail pinch [54]. A self-manufactured FUS transducer (fundamental frequency = 0.7 MHz) was used. For ultrasound sonication, burst signals of 0.7 MHz were generated by a function generator (A33420; Agilent, Santa Clara, CA, USA) and amplified by a radiofrequency power amplifier (240L; E&I, NY, USA). Acoustic pressure was measured in a free field filled and rat skull bone with deionized degassed water using a needle-type hydrophone (HNA-0400; ONDA, Sunnyvale, CA, USA) to estimate the transcranial pressure and acoustic deformation [55]. The diameter and length of the half-maximum pressure amplitude of the ultrasound field and transcranial ultrasound field were within 2 and 2 mm, respectively (Figure 7B,C). During sonication, the ultrasonic gel was used to coat the tFUS inducer of the animal’s head, and the sonication region was aimed at the left primary motor cortex (Figure 7D).

**Figure 8:**
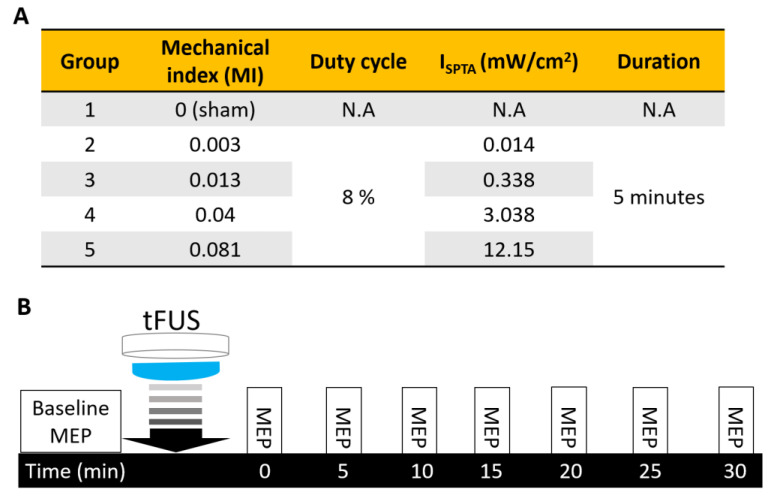
(**A**) Summary of tFUS parameters used in individual experimental groups. MI: Mechanical index; I_SPTA_ = spatial-peak temporal averaged intensity. Duty cycle = percentage of time divided by the total time of a stimulus train. (**B**) Motor-evoked potential (MEP) recording and tFUS sonication protocol.

The ultrasound exposure parameters of the groups and experimental design flow are summarized in Figure 8A. tFUS exposure level was set from 0 mechanical index (MI) to 0.08 MI in groups 2 to 5. The duty cycle was set at 8% (pulse repetition frequency = 100 Hz; burst length = 0.8 ms), which produced weak exposure intensities (I_SPTA_ from 0.014 mW/cm^2^ to 12.15 mW/cm^2^). The total sonication duration was 5 min. Figure 8B illustrates the study protocol and time course. Initially, MEP was recorded for 10 min to serve as the baseline, and tFUS was then performed while the animals were under anesthesia. MEP recording continued for 30 min to measure the post-tFUS effects.

### 4.3. Recordings and TMS Assessments

The assessment of changes in MEP using TMS is a common measurement tool in clinical practice involving cortical neuromodulation of the motor cortex [54,57]. Electromyographic activity (EMG) was recorded with monopolar uninsulated 27G stainless steel needle electrodes inserted into the belly of the bilateral brachioradialis muscle [33,54]. A reference electrode was positioned distally in the paw [33,54]. The EMG signal was amplified (1000×) and filtered using a 60-Hz notch and 10 Hz–1 kHz bandpass filters before digitization at 4 kHz (MP36, BIOPAC System, CA, USA) [54].

All TMS evaluation sessions were performed using a Rapid^2^ magnetic stimulator (Magstim, Whitland, UK) and a figure-of-eight coil (external diameter = 55 mm, internal diameter = 10 mm; Magstim). To evaluate the effect of different FUS schemes on cortical excitability, MEPs were recorded using single-pulse TMS. tFUS sonication was performed on the left M1 region. On the basis of these results, we investigated the effect of weak tFUS using ipsilateral sonication input of the brain. The coil was held in the stereotaxic frame with a junction of rings placed over the rat’s dorsal scalp; this is a position that can reliably elicit equally bilateral forelimb MEPs [54,58]. MEPs evoked using single-pulse TMS were recorded to evaluate the effect of tFUS on cortical excitability. The resting motor threshold (RMT) is defined as the minimal intensity of stimulation required for eliciting MEPs from a contralateral brachioradialis muscle of > 20 μV in 5 out of 10 consecutive trials [54,59]. For the assessment of MEP amplitudes, single TMS pulses were applied at 10-s intervals at 120% RMT, and the EMG signal was recorded for 200 ms after a single TMS pulse. The stimulus intensity remained unchanged throughout the experiment. Peak-to-peak amplitudes of MEP were analyzed offline. To compare the effect of interventions on cortical excitability, all averaged MEP amplitudes were normalized to baseline before FUS intervention.

### 4.4. Histological and Immunohistochemistry Examinations

To confirm the safety of protocols, rats received I_SPTA_ 50 mW/cm^2^ tFUS under high tFUS stimulation intensity and were sacrificed 90 min after receiving tFUS for histological investigation of the brain tissue. Immunohistochemical analysis was used to detect the expression of the GFAP, which indicates the activation of astrocytes following injury or stress [48,60,61,62,63]. Brain sections (30 µm) were cut free-floating in phosphate-buffered saline using Leica CM3050 S Research Cryostat (Leica Biosystems, Nussloch, Germany) for each tissue block, and five consecutive sections per well were collected in multiwell culture plates. Immunohistochemical staining was performed on formalin-fixed, frozen sections using rabbit polyclonal anti-GFAP antibody (ab7260; dilution 1:1000; Abcam, Cambridge, UK) as the primary antibody. To investigate the mechanism of neuromodulation from various weak tFUS intensities on the motor cortex in brain tissue, rats from group 1 (sham) to group 5 (I_SPTA_ = 12.15 mW/cm^2^) were sacrificed 20 min after receiving tFUS. C-Fos (using anti-c-Fos [1:1000, AB190289; Abcam]) [64] and GAD-65 (using anti-GAD-65 [1:100, GAD-65-101AP; FabGennix, Frisco, TX, USA]) [37,65] were used to examine expression level changes in the motor cortex. Changes in GFAP-positive signals with FUS sonication were quantified, and the number of c-Fos-positive and GAD-65-positive cells were analyzed using Image J software (Media Cybernetics, Rockville, MD, USA).

### 4.5. Primary Neuron Cell Culture and Real-Time Calcium Signal following Weak-Intensity Sonication

In addition to assessing the neuromodulatory effects of tFUS in vivo, we examined the changes in neural activity under weak sonication in vitro in the primary neurons derived from rodent embryonic E14-15. The forebrain was isolated from embryonic E14-15 under a dissecting microscope. The forebrain tissue was digested with papain (1 mg/mL in Ca^2+^, Mg^2+^-free Hanks’ balanced salt solution [HBSS]; HBSS w/o Ca^2+^-Mg^2+^: 5.33 mM KCl, 0.44 mM KH_2_PO_4_, 137.93 mM NaCl, 0.34 mM Na_2_HPO_4_, 8.34 mM NaHCO_3_, 5.56 mM glucose, pH 7.3, and 300 mOSM) at 37 °C, gently shaken for 20 min, mechanically minced with a glass pipette, and then centrifuged at 100× *g* for 5 min. The clear supernatant was discarded, and cell pellets were resuspended in Ca^2+^, Mg^2+^-free HBSS. Cells were cultured at 10^6^ cells/mL in 35-mm glass bottom dishes (ibidi, Martinsried, Germany), which were coated with poly-L-lysine and supplemented with Neurobasal medium containing B27 (2%; Thermo Fisher, Waltham, MA, USA), GlutaMax (0.5 mM; Thermo Fisher), glutamatic acid (25 μM; Thermo Fisher), and penicillin–streptomycin (1%; GeneDirexX) at 37 °C /5% CO_2_ in a humidified incubator. Primary neurons were used for experiments after 11–14 days of culturing culture. Before the observation of calcium images, primary neurons were loaded with fluorescent calcium indicator Fluo-4-AM (2%; Thermo Fisher) for 20 min at room temperature [66].

A ring-shaped FUS transducer was used in the cultured dish during calcium imaging. The transducer was placed confocally with the objective of the confocal fluorescence microscope (LS780, Carl Zeiss, Oberkochen, Germany). The culture dish was filled with HBSS and covered with the ultrasound transducer. The FUS intensity applied for observation was the same as that used for group 5 (MI = 0.08, duty cycle = 8%, I_SPTA_ = 12.15 mW/cm^2^, duration = 5 min). During the experiment, an ultrasound was performed on primary neurons for 5 min, and then the real-time neural calcium signals were recorded for 20 min.

To confirm the modulated neural activities using ultrasound, a piezo inhibitor, GsMTX-4 [67] was freshly diluted with distilled water, and 5 μmol/L (piezo inhibitor) was added to cultures 30 min before ultrasound treatment. After sonication, Fluo-4 in the primary neurons was first fixed with 1-ethyl-3-(3-dimethylaminopropyl) carbodiimide (40 mg/mL, EDAC; Sigma-Aldrich, St. Louis, MO, USA) for 30 min. Subsequently, the primary neurons in culture dishes were fixed using 4% formaldehyde for 20 min at room temperature. The primary and second antibodies used for immunostaining to examine activated GABAergic neurons from weak ultrasound stimulation were rabbit anti-GABA (1:2000; Sigma-Aldrich) and Alex Fluor 488-conjugated goat-anti-rabbit igG (1:1000, Abcam), respectively.

### 4.6. Statistical Analysis

All data are expressed as the average ± standard error of the mean (SEM). Data were analyzed using SPSS for Windows version 17.0 (IBM, Armonk, NY, USA). Comparisons of the tFUS-induced changes in MEPs, which were normalized to the last 5-min pre-FUS baseline, were performed using two-way repeated-measures analysis of variance (ANOVA) with sonication (from I_SPTA_ 0 [sham] to 12.15 mW/cm^2^) as a between-subject factor and time (5, 10, 15, 20, 25, and 30 min after FUS) as a within-subject factor. The data of GFAP immunohistochemistry, c-Fos, and GAD-65 were analyzed using a one-way ANOVA followed by Dunnett’s post hoc comparisons.

## Data Availability

The raw data supporting the conclusion of this study are available from the corresponding authors on reasonable request.

## Supplementary Materials

The following supporting information can be downloaded at: https://www.mdpi.com/article/10.3390/ijms24032578/s1, Figure S1: Resting motor threshold (RMT) in each group. Data are presented as the mean ± standard error of the mean (n = 15 per group). Figure S2: Representative images of Fluo-4 and anti-GABA immunostaining of rodent primary cortical neurons at DIV 12 after weak ultrasound stimulation. Weak ultrasound stimulation–activated neurons were stained with Fluo-4 (green). The activated neurons were partially co-immunostained with anti-GABA.
