# Supplementary material for: Weak Ultrasound Contributes to Neuromodulatory Effects in the Rat Motor Cortex
Source: Int J Mol Sci. 2023 Jan 30;24(3):2578. doi: 10.3390/ijms24032578 (PMC9917173; doi:10.3390/ijms24032578)
Supplement: Supplementary file 1 [file ijms-24-02578-s001.zip › ijms-2116595-supplementary.pdf]

Supplementary Figure

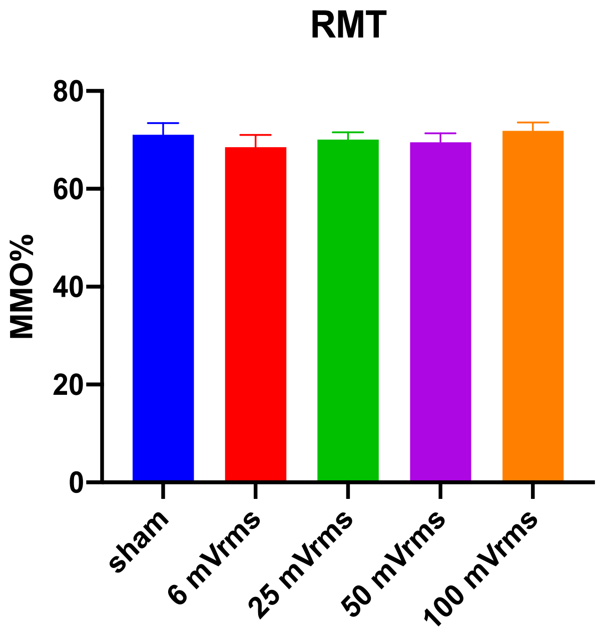

Supplementary Figure S1. Resting motor threshold (RMT) in each group. Data are presented as the mean ± standard error of the mean (n = 15 per group).

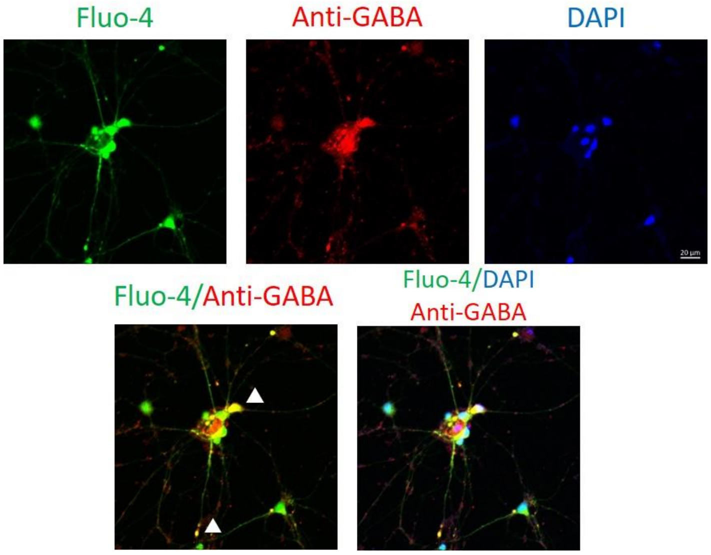

Supplementary Figure S2. Representative images of Fluo-4 and anti-GABA immunostaining of rodent primary cortical neurons at DIV 12 after weak ultrasound stimulation. Weak ultrasound stimulation–activated neurons were stained with Fluo-4 (green). The activated neurons were partially co-immunostained with anti-GABA.
